# Understanding service user-defined continuity of care and its relationship to health and social measures: a cross-sectional study

**DOI:** 10.1186/1472-6963-12-145

**Published:** 2012-06-08

**Authors:** Angela Sweeney, Diana Rose, Sarah Clement, Fatima Jichi, Ian Rees Jones, Tom Burns, Jocelyn Catty, Susan Mclaren, Til Wykes

**Affiliations:** 1 Mental Health Sciences Unit, University College London, London, UK; 2 Department of Health Service and Population Research, Institute of Psychiatry, King’s College London, London, UK; 3 Department of Biostatistics, Institute of Psychiatry, King’s College London, London, UK; 4 School of Social Sciences, Bangor University, Bangor, UK; 5 Department of Psychiatry, University of Oxford, Oxford, UK; 6 Department of Mental Health, St Georges, University of London, London, UK; 7 Faculty of Health and Social Care, London South Bank University, London, UK; 8 Department of Psychology, Institute of Psychiatry, King’s College London, London, UK

**Keywords:** Service users, Continuity of care, Health and social outcomes

## Abstract

**Background:**

Despite the importance of continuity of care [COC] in contemporary mental health service provision, COC lacks a clearly agreed definition. Furthermore, whilst there is broad agreement that definitions should include service users’ experiences, little is known about this. This paper aims to explore a new construct of service user-defined COC and its relationship to a range of health and social outcomes.

**Methods:**

In a cross sectional study design, 167 people who experience psychosis participated in structured interviews, including a service user-generated COC measure (CONTINU-UM) and health and social assessments. Constructs underlying CONTINU-UM were explored using factor analysis in order to understand service user-defined COC. The relationships between the total/factor CONTINU-UM scores and the health and social measures were then explored through linear regression and an examination of quartile results in order to assess whether service user-defined COC is related to outcome.

**Results:**

Service user-defined COC is underpinned by three sub-constructs: preconditions, staff-related continuity and care contacts, although internal consistency of some sub-scales was low. High COC as assessed via CONTINU-UM, including preconditions and staff-related COC, was related to having needs met and better therapeutic alliances. Preconditions for COC were additionally related to symptoms and quality of life. COC was unrelated to empowerment and care contacts unrelated to outcomes. Service users who had experienced a hospital admission experienced higher levels of COC. A minority of service users with the poorest continuity of care also had high BPRS scores and poor quality of life.

**Conclusions:**

Service-user defined continuity of care is a measurable construct underpinned by three sub-constructs (preconditions, staff-related and care contacts). COC and its sub-constructs demonstrate a range of relationships with health and social measures. Clinicians have an important role to play in supporting service users to navigate the complexities of the mental health system. Having experienced a hospital admission does not necessarily disrupt the flow of care. Further research is needed to test whether increasing service user-defined COC can improve clinical outcomes. Using CONTINU-UM will allow researchers to assess service users’ experiences of COC based on the elements that are important from their perspective.

## Background

Continuity of care [COC] is widely considered to be a central goal of contemporary mental health service provision [1,2]. This centrality is largely due to deinstitutionalization and the advent of community care: services that were formerly provided within one institution - such as shelter, activities and psychiatric treatment – have become dispersed amongst a number of agencies. Consequently, the provision of coherent, smooth care has become problematic, and COC has emerged as a central indicator of successful, integrated community services. Furthermore, discontinuities have been linked to adverse outcomes. For example, a number of official inquiries into suicides and homicides by people with psychiatric diagnoses have suggested that a lack of COC may have been a causal factor [3,4].

Whilst there is some evidence that COC positively affects service users’ outcomes [5-7], such relationships have not been uncovered consistently [8]. Moreover, efforts to research COC and its relationship to outcomes have been hampered by a lack of an agreed definition; COC has been described as “a conceptually underdeveloped, vague and over-inclusive construct lacking a solid empirical foundation” [9]. Individual research teams have typically defined continuity for their specific research, resulting in sporadic and disconnected measurement. Consequently, there is “virtually no consistency in the way that continuity of care has been measured or in the choice of outcome measures” [5].

In recent years, there has been a growing consensus that COC is a multi-dimensional construct that should centralise service users’ experiences [5,10]. However, existing measures tend to assess a single dimension of COC, such as hospital discharge, and either ignore service users’ definitions [11] or conflate them with those of staff [12,13]. Consequently, little is known about service users’ views of, priorities for or experiences of COC [14], and, “it is not definitively known whether program interventions and administrative policy changes intended to facilitate COC are actually experienced as such by patients” [15]. Having the means to measure continuity of care from a service user perspective will enable researchers to establish in the first instance whether there is a relationship between service user-defined COC and service users’ outcomes. If evidence suggests that such a relationship exists, the specific elements of COC that are most predictive of positive outcomes can be identified. From this, interventions can be designed that target the elements of COC that are most important to service users and most likely to improve their outcomes and experiences of mental health services.

Because of the need for a service user focussed measure of COC, in an earlier study our research team used a participatory model to generate a measure of service users’ experiences of continuity of care, CONTINU-UM (CONTINU-ity of Care – User Measure) [16]. We found that service user-defined COC overlaps with existing academic-led conceptualisations but also differs: some components are reconceptualised from the perspective of receiving rather than delivering services and three components are not found elsewhere in the COC literature; these are peer support, day centres and avoiding services [17]. Thus, service user-defined COC is a unique conceptualisation of the construct.

CONTINU-UM was employed as a measure of experienced COC in a larger research programme on continuity of care (Experiences of Continuity of care and Health and social Outcomes (ECHO) [18]. Whilst the current paper relates only to participants who completed CONTINU-UM, a separate study in the ECHO research programme conducted exploratory factor analysis with all participants (regardless of whether or not they had completed CONTINU-UM) in order to understand how different components of continuity of care related to one another. [19] Seven independent factors were identified using principle component analysis and varimax rotation. CONTINU-UM formed a factor along with service users’ therapeutic alliances, their proportion of met needs and service user-initiated breaks in care. This was the largest factor and was named *Experience and Relationship*. It was associated with better quality of life and fewer current symptoms but an increase in symptoms the following year. However, directions of causality were unclear and it was concluded that, “Further work is needed to identify the central continuity factors for establishing high-quality care for people with chronic mental health problems”.

Our analysis complements and extends these findings by specifically exploring the relationship between health and social outcomes and the factors that make up user-defined continuity of care, as measured by CONTINU-UM. Our first hypothesis is that as COC should lead to improved outcomes then better COC will be related to a greater proportion of met needs; fewer symptoms; enhanced quality of life; greater feelings of empowerment; and better relationships with staff. Secondly, COC can be most seriously compromised when service users experience transitions between services, and in particular when admitted to hospital. Therefore we explore how experiencing a hospital admission affects COC and health and social outcomes. Finally, we will try to identify whether a group of service users exists who may be vulnerable to ‘falling through the gap’ of care by examining the quality of life and symptom severity of people with the poorest continuity of care.

## Methods

### Setting, sample and procedures

Service users were recruited from seven Community Mental Health Teams (CMHTs) in two South London NHS Trusts. CMHTs were situated in two inner-city areas with high Jarman indices and a settled, suburban area with a lower Jarman index in order to recruit service users with widely varying socio-demographic characteristics. All service users eligible for recruitment were invited to participate with written consent gained prior to interviewing. The inclusion criteria were selected to ensure that participants had experiences of COC: (a) diagnosis of psychosis for at least two years; (b) aged 18–65; (c) in contact with services for at least two years; (d) on the caseload of a CMHT for at least six months; and (e) on enhanced care programme approach. This is the group of service users who have been identified as likely to be most in need of continuity of care [1] due to ongoing and episodic needs [20] that can require multiple interventions at different locations either at the same point in time or over time [13]. This group can also experience crises that lead to hospitalisation, placing emphasis on the need for continuity during transitions between hospital and the community, and for services to vary rapidly. Therefore, exploring continuity of care with this group in the first instance was felt to be necessary and appropriate.

Service users provided socio-demographic and service contact information and participated in structured quantitative interviews consisting of a range of measures; these measures are described below.

Ethics approvals were granted by South London and Maudsley/Institute of Psychiatry Ethics Committee (reference 128/01) and Wandsworth Research Ethics Committee (reference 01.42.8).

### Measures

All measures were completed by service users in interviews.

***CONTINU-UM*** (CONTINUity of care – User Measure) contains sixteen domains (see [17] for specific development). Qualitative methods were used to generate and validate these domains. Briefly, five focus groups were each held twice with service users who matched inclusion criteria a-c outlined above. Groups discussed their experiences of mental health services and definitions of and priorities for COC. Thematic analysis was used to identify priority elements of continuity of care. The research team then constructed a draft measure which was revised by two Expert Panels of service users. Finally, a small consultation exercise with two COC researchers and one service user researcher led to final revisions. A full description of the methods used to develop CONTINU-UM can be found in [16] and [17]. The final domains of CONTINU-UM are: access; range; waiting; out of hours support; hospital discharge; staff changes; information; flexibility; individual progress; day centres; care plans; crisis systems; staff communication; peer support; life histories; and avoiding services. Each domain contains three five point adjectival scales for the importance of (***a***); experience of (***b***); and satisfaction with (***c***) COC domains. All sub-scales are internally consistent (Cronbach’s alphas are 0.75 for ***a*** items, 0.74 for ***b*** items and 0.88 for ***c*** items) and the measure has good test retest reliability [17]. All analyses presented here are conducted on ***b*** items. Three items were excluded because they are not applicable to all participants: day centres, hospital discharge and care plans. Item details can be found in Table 1. The possible range of scores is 17 to 85 with a high score meaning high continuity of care.

**Table 1:** Prorated factor analysis results for the three factor solution with orthogonal rotation

**Item**	**Definition**	**Factor 1: preconditions**	**Factor 2: staff related-continuity**	**Factor 3: care contacts**
Access	Getting the services you need when you need them	0.7959		
Range	Getting the range of services you need	0.8281		
Waiting	Waiting for the services you need		0.3295	−0.4877
Out of hours	Getting support from services outside of office hours			0.6358
Staff changes	Seeing the same staff		0.7583	
Information	Getting the information you need from staff	0.5824	0.3300	
Flexibility	Having levels of support change as your needs change		0.6292	
Individual progress	Having services that help you to progress	0.6755		
Crisis	Having agreed crisis plans			0.5207
Staff communication	Staff tell each other what is happening	0.3830	0.5679	
Peer support	Receiving support from other people who use services			0.3599
Life histories	Explaining yourself to new staff members each time		0.3974	
Avoiding services	Choosing when you see services			0.4912
**Cronbach’s Alpha**^**1**^		**0.73**	**0.52**	**0.32**

^1^ Cronbach’s alphas were calculated for final assigned factor items, and not all loading items.

***Camberwell Assessment of Need (CAN)*** is a self-report instrument measuring perceived needs in 22 areas such as health, social care, functioning and service receipt [21]. The psychometric properties of the CAN are well established [22]. The proportion of met needs was calculated as the percentage of total needs that were met. Not applicable responses and missing responses did not contribute to the total score. The range of scores for the CAN is 0 to 100, with a higher score suggesting a greater number of met needs.

***Scale to Assess the Therapeutic Relationship in community mental health care*(STAR) – service user version** assesses the relationship between the service user and their care co-ordinator, psychiatrist or other nominated professional. It is a relatively new instrument which has been found to have satisfactory psychometric properties [23]. The STAR score used in the analysis was the rating of a participant’s relationship with their care co-ordinator (total score). Where this score was missing the psychiatrist total score was used, and where this was missing, the total score for other significant professional was used. The range of scores for the STAR is 0 to 48 with a higher score suggesting a better therapeutic relationship.

***Brief Psychiatric Rating Scale*(BPRS)** assesses psychiatric symptoms in five main areas: anxiety and depression, anergia, thought disturbance, activation and hostility–suspiciousness [24]. The BPRS is a well-established and heavily used scale with high reliability and validity [25]. The BPRS generates a total score ranging from 18–126 with a higher score suggesting a higher level of symptoms.

***Schedule for the Evaluation of Individual Quality of Life (*SEIQoL)** is a self report instrument that measures a person’s satisfaction with his or her quality of life in five self-identified areas. A review of 39 published studies which used SEIQoL found evidence of feasibility, convergent validity and test retest reliability [26]. SEIQoL generates a total score ranging from 0–100 with a higher score meaning greater quality of life [27].

***Boston User Empowerment Scale (BUES)*** rates empowerment, as defined by service users. Psychometric testing has generated evidence of internal consistency and validity [28]. It generates a total score between 28 and 112 with a higher score meaning greater empowerment.

### Statistical analyses

All statistical analyses were carried out in Stata 11. For CONTINU-UM, STAR, BPRS and BUES, total scores were pro-rated where there were 10% or less missing items. Pro-rating is a way of producing total scores when some items are missing; if a person had 90% or more data available, an average of their available data was taken and their missing values were replaced by this average. From this, a total score was calculated for each person. If there were more than 10% missing items, the score was recorded as missing. Missing SEIQoL data were not pro-rated but were recorded as missing. All scores were standardised (by dividing scores by their standard deviations).

#### Exploration of service-user defined continuity of care

Data were explored to ensure suitability for factor analysis. Eigenvalues, scree plots and amount of common variance explained were used to help determine the number of factors in the model. Factor rotation aimed to achieve simple structure, making the solutions more likely to be replicable and interpretable, with item loadings greater than 0.3 accepted. Where the same item loaded on two or more different factors, a decision was made as to where the item would be placed based on conceptual fit and loading. Standardised total and factor scores were used in all analyses (created by dividing scores by their standard deviations).

#### The relationship between service-user defined continuity of care and health and social measures, including hospital admission

Separate regression analyses were used to assess the relationships between CONTINU-UM total and factor scores (the dependent variables), health and social measures (proportion of met needs, CAN; therapeutic alliances, STAR; symptoms, BPRS; quality of life, SEIQoL; and empowerment, BUES) and hospital admissions in the previous 12 months (binary yes or no). For the latter analysis, socio-demographic variables found to be related to hospital admission were included as fixed covariates.

Regression analysis was also used to explore whether the relationship between CONTINU-UM total and factor scores and other health and social care measures varied depending on whether people had been admitted to hospital in the previous 12 months or not (binary yes or no; the dependent variable). The health and social measures, and an interaction between the two, were included as fixed covariates.

Finally, CONTINU-UM, BPRS and SEIQoL quartiles were investigated to determine the proportion of people who had low continuity, high symptoms and poor quality of life.

## Results

### Participant profile

180 participants were recruited to the study and asked to complete all measures. This was 36% of all eligible CMHT service users and was considered an adequate sample size [19]. 167 of the 180 sample completed CONTINU-UM. The participant profile and measure scores can be found in Table 2. No socio-demographic data were found to be related to continuity of care, as assessed by CONTINU-UM.

**Table 2:** Summary of participant characteristics

**Characteristic**	**Category**	**Frequency (%)**
Sex	Male	93 (55.7)
	Female	74 (44.3)
Ethnicity	White	113 (67.7)
	Asian/Asian British	15 (9)
	Black/Black British	31 (18.6)
	Mixed heritage	6 (3.6)
	Other	2 (1.2)
Living situation	Living alone	66 (39.5)
	Living with partner	27 (16.2)
	Living with parents	18 (10.8)
	Living with relatives	10 (6.0)
	Living with others	46 (27.5)
Education	Up to age 16	64 (38.3)
	Above 16	103 (61.7)
Employment	Full time	7 (4.2)
	Part time	9 (5.4)
	Sheltered scheme	1 (0.6)
	Student	5 (3.0)
	Retired	8 (4.8)
	Seeking work	11 (6.6)
	Unable to work	81 (48.5)
	Other	4 (2.4)
	Missing	41 (24.6)
Hospital admission over past year?	Yes	57 (34.1)
	No	106 (63.5)
	Missing	4 (2.4)
Total number of hospital admissions	None	11 (6.6)
	1-5	113 (67.7)
	6-10	30 (18.0)
	11+	13 (7.8)
	**Mean (sd)**	**Median**
Age^1^	43.6 (10.8)	44.0
CONTINU-UM score^2^	39.2 (9.3)	40.0
CAN score^3^	74.0 (28.3)	81.8
BPRS score^4^	32.5 (10.2)	31.0
BUES score^5^	76.6 (8.0)	76.0
STAR score^6^	37.0 (8.7)	39.0
SEIQoL score^7^	62.3 (16.6)	64.7

^1^ The age range was 19 to 65.

^2^ CONTINU-ity of Care – User Measure. The range was 14 to 59.

^3^ Camberwell Assessment of Need. The range was 0 to 100.

^4^ Brief Psychiatric Rating Scale: The range was 18 to 74.

^5^ Boston User Empowerment Scale. The range was 54 to 99.

^6^ Scale to Assess the Therapeutic Relationship in community mental health care. The range was 6 to 48.

^7^Schedule for the Evaluation of Individual Quality of Life. The range was 22 to 100.

#### Exploration of service-user defined continuity of care

An examination of the data indicated that they were appropriate for exploration through Principle Components Factor Analysis: the correlation matrix suggested interrelationships among items, individual measures of sampling adequacy ranged from 0.76 to 0.95, the KMO statistic was 0.9 and Bartlett’s test of sphericity was significant, chi square = 347.872, df = 78, p < 0.001. Factor analysis produced eigenvalues ranging from 3.12 to 0.32 with extracted item communalities ranging from 0.31 to 0.80. The scree plot suggested that two, three, or four factor models could represent the constructs underlying service user-defined COC. The three factor model with orthogonal rotation (varimax) produced the most interpretable factor structure, with all items contributing to the model.

The factor analysis results are shown in Table 1. The first factor, named preconditions for COC, consisted of access, range, information and individual progress. These items were internally consistent. Staff communication loaded between 0.3 and 0.4, and was assigned to factor two where it had a higher loading and better conceptual fit. Thus, *preconditions for COC* is defined as easy access to a range of needed services accompanied by high quality information and having the services that are needed to move forward. The second factor, named staff-related COC, consisted of staff changes, flexibility, staff communication and life histories. These items showed moderate internal consistency. Waiting and information loaded between 0.3 and 0.4, and were assigned to factors where the factor loadings were higher and conceptual fit was greater. S*taff-related COC* is therefore defined as good communication between staff and infrequent staff changes meaning that service users don’t have to repeat their life histories to new staff, and flexible service responses. Finally, the third factor consisted of waiting (negative loading), out of hours support, crisis, peer support and avoiding services, and was named care contacts. Therefore, c*are contacts* is defined as waiting for services, being able to choose to avoid services and having support from peers, out of hours and through established crisis systems. This factor had low internal consistency.

#### The relationship between service-user defined continuity of care and health and social measures, including hospital admission

Greater overall COC (total CONTINU-UM score) was related to better therapeutic alliances and a greater proportion of met needs across the majority of groups and scores tested (see Table 3). Both the *preconditions for COC* factor and *staff-related COC* factor showed similar relationships. However, the *care contacts* factor showed no relationships with the health and social measures.

**Table 3:** Regression of CONTINU-UM scores and health and social measures for all participants (standardised regression coefficients, 95% Confidence Interval, p-value, N)

**Health and social measures**	**Total score**	**Preconditions score**	**Staff related continuity score**	**Care contacts score**
CAN proportion of met needs	0.43	0.44	0.27	0.13
	0.29, 0.57	0.29, 0.58	0.12, 0.42	−0.03, 0.28
	<0.01	<0.01	<0.01	0.11
	160	162	160	160
STAR total score	0.34	0.41	0.24	−0.03
	0.19, 0.50	0.26, 0.56	0.08, 0.40	−0.20, 0.13
	<0.01	<0.01	<0.01	0.70
	147	148	147	147
BPRS total score	−0.12	−0.24	−0.04	0.06
	−0.27, 0.04	−0.39, -0.09	−0.20, 0.11	−0.10, 0.22
	0.14	<0.01	0.58	0.44
	160	162	160	160
SEIQoL total score	0.10	0.17	0.003	0.03
	−0.07, 0.27	−0.003, 0.33	−0.17, 0.18	−0.14, 0.20
	0.25	0.05	0.97	0.74
	134	135	134	134
BUES total score	−0.05,	0.09	−0.16	−0.08
	−0.21,0.11	−0.07, 0.25	−0.31, 0.002	−0.24, 0.08
	0.57	0.26	0.05	0.30
	154	156	154	154

*Preconditions for COC* was additionally associated with having increased quality of life and fewer symptoms whilst *staff-related COC* was additionally related to empowerment (BUES).

The only demographic variable related to hospital admission was age, with younger people more likely to have had an admission in the previous 12 months (mean age with no admission = 46 year, with an admission = 39 year, t = −4.35, df163, p < 0.001). This variable was therefore included as a fixed covariate in this regression analysis. There was a significant relationship between hospital admission and overall COC, with those who had experienced a hospital admission in the previous 12 months having better COC (standardised regression coefficient = 0.18; p-value = 0.03). However, no such relationship was found for the three factors (p-values = 0.67, 0.19 and 0.32 respectively).

The relationships between COC scores and health and social measures were similar in people who had and had not experienced a hospital admission in the previous 12 months. The only exception to this was quality of life: participants who had experienced a hospital admission in the previous 12 months had a positive correlation between COC and quality of life whilst no such relationship was found for those who had not had an admission. This is also true for staff-related COC scores and quality of life.

The 25th percentile for CONTINU-UM scores was 34, and for quality of life was 51.6. The 75th percentile for BPRS scores was 38. These quartile scores were used to define poor COC, high symptoms and low quality of life in our sample. We found that for those with the poorest COC, 27% (n = 12) had high symptom scores, 20.5% (n = 9) had low quality of life, and 7% had both the highest level of symptoms and lowest quality of life (i.e. fell in the 25th percentile of CONTINU-UM, the 75th percentile of the BPRS and the 25th percentile of SEIQoL).

## Discussion

### Understanding service user-defined continuity of care

Factor analysis identified three constructs underlying service user-defined continuity of care, with all items contributing to the model. The first factor, consisting of access, range, information and individual progress, is suggestive of preconditions or building blocks for COC, as without easy access to a range of services that – crucially – help you to progress, COC cannot be in place. This mirrors experiences described in a preliminary qualitative study in which participants reported that access to needed services (range), particularly during first contact with mental health services, should be rapid and accompanied by high quality information, and that these are among the most important elements of user-defined COC [17]. More broadly, these findings resonate with a small but growing body of service user-led research which has found that service users lack easy access to the services they feel they need [29] as well as lacking information, despite needing to negotiate complex systems [30]. The participants in our qualitative phase felt that without information they were unable to negotiate the mental health system and therefore unable to be the facilitators of their own COC. The burgeoning recovery literature attests to service users’ desire for services that help them move forward, yet this element of COC is rarely found in the prominent COC literature. Whilst information, range and access all appear in the prominent literature as aspects of continuity of care [31], informational continuity is defined as information exchange between professionals [9,14]. Conversely, service users define informational continuity as the flow of adequate information from professionals to service users [17]. This underscores how elements of COC differ when service users are asked to define and prioritise them.

The second factor represents contacts with staff. Assessing the frequency of staff contacts and changes is a common way of understanding and measuring (dis)continuity [14,31,32]. What is new about our approach is that staff contacts have been operationalised from a service user perspective. This has resulted in the inclusion of indicators that are important to service users, most notably the frequency with which they have to repeat their life history to new members of staff. This dimension of COC is rarely found elsewhere in the literature. However, the internal consistency for this factor was moderate, and so some caution needs to be employed in its interpretation. The qualitative development phase of this work revealed that whilst most people felt that frequent staff changes were disruptive, changes were actively wanted where relationships were failing. Thus, whilst relational continuity is important to service users, the quality of relationships is key. Separate strands of this research have found that service users value relationships with professionals but these relationships are fragile and vulnerable to disruption [33], and have identified the workforce factors that can impact negatively on staff-related COC such as high staff turnover and use of temporary and agency staff [34].

Finally, the third factor represents service users’ care contacts. Interestingly, from the direction of factor loadings it appears that service users who had access to peer and out of hours support and had crisis systems in place were *more* likely to experience waits for services and to be able to avoid services. There are a number of possible explanations for this; for example, it may be that people who are experiencing gaps in care are accessing alternative support whilst they wait, or that staff who see service users as well supported are not prioritising their access to mainstream services. Further research is needed to explore this. However, we also found during the qualitative phase that service users consider waiting for services to be acceptable if the person is not approaching crisis. Once again, understanding COC from a service user perspective has expanded the traditional definition of COC from contacts with services alone to contacts with peers [17]. The importance of peer support is a common research finding by service user researchers [29,30,35]. Yet positive and helpful relationships with staff members are also highly valued. For example, a literature review of what service users want from services identified good staff relationships and peer support as among the top priorities [36] and service users who are considered ‘hard to engage’ have stressed the importance of building and maintaining relationships with staff [37]. Therefore, clinicians and researchers should consider a range of formal and informal supports when assessing service users’ experienced continuity of care. One word of caution, however: the internal consistency for this factor is low and so it may not be a reliable summary measure.

### Does service-user defined continuity of care relate to health and social measures?

CONTINU-UM scores clearly relate to independent health and social measures, but the picture is not a simple one. The hypotheses that having service user-defined COC in place would relate to better therapeutic relationships and a greater proportion of met needs were supported across the majority of factors and groups we tested. Having the preconditions or building blocks for COC in place was additionally related to lower symptoms and greater quality of life for the whole group, whilst staff-related COC was additionally related to empowerment. This latter finding suggests that empowerment is related to service users’ experiences of the consistency of staffing and flexibility of service responses. Whilst the directions of causality are unknown, these findings nonetheless highlight the importance of asking service users about the elements of COC that they deem essential. Further research should test whether having the preconditions for COC and staff-related COC in place affects outcomes.

A notable exception to the pattern of relationships was the third factor, care contacts (consisting of waiting, out of hours support, crisis systems, peer support and avoiding services) which demonstrated no relationships to health and social outcomes. Thus, this dimension of continuity of care may be of less importance to outcome. This is a little surprising given the emphasis on care contacts in establishing COC. One explanation may lie in service users’ priorities for continuity of care: none of the elements of continuity that form the care contacts factor were amongst the highest rated elements by service users [17]. Instead, service users rated those elements that constitute preconditions for COC most highly. This suggests that service users prioritise the elements of continuity that are most predictive of positive outcome, and that this excludes care contacts. A further issue is that this factor had low internal consistency and it may be that it is not a reliable summary measure. Whilst low internal consistency could be due to the small number of items within the factor [38], given that care contacts did not demonstrate relationships with health and social measures we suggest that it is not used as a summary measure in future research.

In short, our findings suggest that the central COC factors for establishing high-quality care may be those elements that form the preconditions or building blocks for COC as well as staff-related COC.

### Do interruptions in the flow of care affect continuity of care?

Continuity of care has traditionally been operationalised through hospital admissions and discharges [20,39,40]. Our research has found that having experienced a hospital admission in the previous 12 months was related to an *increase* in COC scores. There are a number of possible explanations for this finding. For example, it may be that hospital admissions and discharges are being managed in a way that promotes rather than disrupts continuity of care, or that those with the highest levels of need are receiving the greatest levels of COC, or indeed a combination of factors. Similarly, a separate study in this research programme found that care coordination (such as having a designated care coordinator) was improved where people had been in hospital the previous year, and that having a greater number of transitions accompanied by documentation (as opposed to undocumented) was linked to experiencing a hospital admission. This latter finding suggests that when service users are admitted to hospital, careful attention is being paid to informational continuity. This supports the interpretation that hospital admissions and discharges are managed in ways that promote COC. However, we also found little difference in the relationship between COC and health and social measures between those who had and had not experienced a hospital admission. The main exception was that those who had been in hospital showed increased quality of life as COC increased. Taken together, these findings suggest that experiencing well managed transitions may have important implications for service users’ quality of life, and that hospital admissions do not necessarily disrupt the flow of care. Additional research is needed to further explore these findings.

Finally, for people with the lowest COC scores, more than one quarter had high BPRS scores whilst a fifth reported low quality of life. Again, the direction of causality is unknown; it may be that service users who are experiencing psychosis begin to disengage with services affecting their quality of life, or that service users with poor continuity of care have poorer quality of life and poorer mental health as a result. In both instances, it is possible that this group of service users could become disengaged from services. Our research suggests that where this occurs, people need easy access to the range of services that they feel would meet their needs to increase engagement and decrease discontinuity.

### Strengths and limitations

This is the first time that a service user-defined measure of COC has been used in such a study. Our sample was large and representative of people for whom there has been a concern about the effects of a lack of continuity of care. Participants were recruited from areas with mixed Jarman indices in a metropolitan context. We believe, therefore, that our findings are generalisable to service users who experience psychosis, are in touch with mental health services and who reside in UK metropolitan areas.

However, only 36% of all eligible CMHT service users participated. It is possible that those who chose not to participate were less well engaged with services, or were perceived by gatekeepers as having poorer continuity of care and therefore not encouraged to participate. In the future, studies should pay close attention to those who are less well engaged and their experienced COC. It may be helpful to explore COC using a service user-defined measure such as CONTINU-UM as this signals the importance of service users’ perspectives to participants and allows the construct to be explored and assessed from their standpoint.

This is a cross sectional study and only explored the relationships of experienced COC with concurrent measures, so we have no evidence of causality. Although there are studies that examine the impact of interventions on continuity of care, the outcomes chosen are rarely those aspects of COC that are prioritised and valued by service users [41]. Our study suggests that increasing service-user defined COC may have implications for met needs and therapeutic alliance which have rarely been shown to be affected by service change. This warrants exploration through further research.

## Conclusions

There is broad agreement that definitions and assessments of continuity of care should include service users’ experiences. This novel study - coupled with previous findings of good test retest reliability and validity for CONTINU-UM [17] - has demonstrated that service user-defined COC is a measurable construct underpinned by three sub-constructs: preconditions, staff-related COC and care contacts. Overall COC and its sub-constructs show a range of relationships with independent health and social measures; such relationships are rarely found in professionally defined measures.

Clinicians have a central role to play in supporting service users to navigate complex systems and in creating the continuity of services, relationships and care contacts that service users feel they need. Further research is needed to establish whether improving COC can impact on the outcomes of care. Future studies should include a measure of service users’ experiences such as CONTINU-UM when exploring continuity of care.

## Competing interests

The authors declare that they have no competing interests.

## Authors’ contributions

AS conducted some analyses, contributed to data interpretation and wrote the first draft of this paper. FJ conducted the majority of statistical analyses. TW contributed to study design, data analysis, data interpretation and writing. JC oversaw data collection and contributed to study design and writing. DR, SC, IRJ, TB and SMcL contributed to study design and writing. All authors read and approved the final manuscript.

## Authors’ information

AS and DR identify as service user researchers and conducted the research from that standpoint.

## Pre-publication history

The pre-publication history for this paper can be accessed here:

http://www.biomedcentral.com/1472-6963/12/145/prepub
